# Nicotine Dependence and Risk of Postpartum Psychosis: A Large Real-World Cohort Study

**DOI:** 10.7759/cureus.114570

**Published:** 2026-08-15

**Authors:** Jessica Kuo, Katelyn D Le, Keanna Gomez, Xuezhi Jiang

**Affiliations:** 1 Psychiatry, Drexel University College of Medicine, Philadelphia, USA; 2 Obstetrics-Gynecology, Drexel University College of Medicine, Philadelphia, USA; 3 Obstetrics-Gynecology, Reading Hospital, Tower Health, Reading, USA

**Keywords:** nicotine dependence, post-partum depression, post-partum psychosis, smoking tobacco, trinetx

## Abstract

Background

Postpartum psychosis is a rare but severe mental health emergency that occurs within 60 days of delivery. Although nicotine is a well-established risk factor for psychosis in the general population, its potential association with postpartum psychosis remains underexplored. Prior research has shown that maternal smoking is associated with an increased risk of postpartum depression. Building on this evidence, our study aims to investigate whether nicotine dependence similarly increases the risk of postpartum psychosis.

Methods

A retrospective cohort study was conducted using the TriNetX database to identify females aged 15-45 with documented deliveries. Two cohorts were formed based on the presence or absence of nicotine dependence during pregnancy. The primary outcome was postpartum psychosis up to 60 days after birth. Risk differences and risk ratios (RRs) with 95% confidence intervals (CIs) were calculated to compare incident postpartum psychosis; Kaplan-Meier survival curves and log-rank tests were used to assess time to event. Hazard ratios (HRs) were estimated using Cox proportional hazards regression. Propensity score matching was conducted to balance demographics and pre-existing mental, behavioral, and neurodevelopmental disorders between cohorts.

Results

The nicotine dependence and control cohorts comprised 155,280 and 1,291,177 patients, respectively. After propensity matching, 154,123 women were included in each group. Postpartum psychosis occurred in 158 patients (0.11%) in the nicotine-exposed cohort and 70 patients (0.05%) in the matched control cohort (p=0.001). Despite the increased relative risk, the absolute incidence of postpartum psychosis remained low in both cohorts. Nicotine dependence was associated with a significantly increased risk of postpartum psychosis (RR (95% CI) = 2.29 (1.73-3.04)). Survival analysis demonstrated a higher hazard of postpartum psychosis among nicotine-exposed patients (hazard ratio 2.27, 95% CI 1.71-3.01; log-rank p < 0.001).

Conclusion

Nicotine dependence is associated with increased risk of postpartum psychosis within 60 days after delivery. These findings suggest that nicotine exposure may represent a clinically relevant risk factor for postpartum psychosis. Further studies incorporating behavioral and socioeconomic data are warranted to clarify underlying mechanisms and inform targeted prevention strategies.

## Introduction

Postpartum psychosis is a rare but severe psychiatric emergency that typically presents within the first several weeks following childbirth. Unlike postpartum depression, postpartum psychosis is characterized by hallucinations, delusions, mood disturbance, and cognitive impairment. Postpartum psychosis carries a high risk of maternal suicide and infanticide and almost always requires urgent psychiatric hospitalization [1]. Despite its low incidence, estimated at approximately 0.9 to 2.6 cases per 1,000 births, postpartum psychosis is associated with substantially greater morbidity than other perinatal mood disorders, including postpartum depression and postpartum blues [1,2].

Established risk factors for postpartum psychosis include a personal or family history of bipolar disorder, prior psychotic illness, and primiparity [1]. However, potentially modifiable risk factors remain poorly characterized. Nicotine use is highly prevalent among reproductive-age women, and nicotine dependence has been consistently associated with psychotic disorders in the general population [3]. In the perinatal context, nicotine dependence before and during pregnancy has been well established as a risk factor for postpartum depression [4-6]. Whether nicotine dependence specifically increases the risk of postpartum psychosis, however, has received little attention.

Given the profound clinical consequences of postpartum psychosis and the widespread prevalence of nicotine use, identifying modifiable risk markers is of critical importance. To our knowledge, no large population-based studies have evaluated the association between nicotine dependence and postpartum psychosis. In this study, we used a large, nationally representative electronic health record database to investigate whether nicotine dependence is associated with an increased risk of postpartum psychosis during the early postpartum period.

## Materials and methods

Data source

We conducted a retrospective cohort study using the TriNetX Research Network, a federated, cloud-based platform that aggregates de-identified electronic health record data from large healthcare organizations across the United States. As of January 2026, TriNetX included records from over 205 million patients across 170 healthcare organizations, with longitudinal data primarily dating back to 2006. The database contains comprehensive clinical information, including demographics, diagnoses, procedures, medications, and laboratory values. All analyses were performed within the secure TriNetX environment. Because the data are fully de-identified and Health Insurance Portability and Accountability Act (HIPAA) compliant, this study was exempt from institutional review board approval.

Population

Using the TriNetX platform, the inclusion and exclusion criteria were outlined using ICD-10-CM codes. All patients included were females aged 15-45 years old, with a documented delivery. Cohort 1 consisted of patients with a recorded delivery (ICD-10-PCS: 10E) and a diagnosis of either nicotine dependence (ICD-10-CM: F17) or tobacco use disorder complicating pregnancy, childbirth, or the puerperium (ICD-10-CM: O99.33). Cohort 2 consisted of patients with a recorded delivery (ICD-10-PCS: 10E) and no diagnosis of nicotine dependence (ICD-10-CM: F17) or tobacco use disorder complicating pregnancy, childbirth, or the puerperium (ICD-10-CM: O99.33) [7]. The index event was defined as the first qualifying delivery, only including events that occurred up to 20 years ago. Patients whose index event occurred 20 years or more ago were excluded. For the smoking cohort, the index event additionally required documentation of nicotine dependence or tobacco use disorder complicating pregnancy. For the control cohort, the index event was defined as the first delivery alone. 

Study design

The primary outcome was postpartum psychosis. It was defined by the presence of any of the following ICD-10-CM diagnoses: puerperal psychosis (F53.1), brief psychotic disorder (F23), or schizophrenia spectrum and other non-mood psychotic disorders (F20-F29). Schizophrenia spectrum diagnoses were included to capture clinically coded postpartum psychosis presentations that may not be labeled explicitly as puerperal psychosis in administrative data. These outcomes were assessed within a time window of 1 to 60 days after delivery. Patients with a qualifying outcome diagnosis prior to the beginning of the outcome window were excluded from the outcome analyses. Propensity score matching was performed on 16 characteristics, including current age, age at index, sex, ethnicity, race, and mental, behavioral, and neurodevelopmental disorders (ICD-10-CM F01-F99). Cohorts were matched in a 1:1 ratio, resulting in 154,123 patients in each cohort.

Statistical methods

Statistical analyses were performed using the TriNetX “Compare Cohorts” tool to evaluate outcomes between cohorts. Measures of association included risk, risk difference, and risk ratio. Time-to-event analyses were conducted using Kaplan-Meier survival curves with log-rank testing and hazard ratios. The proportional hazards assumption was tested using Schoenfeld residuals. Statistics were generated within the TriNetX internal analytics platform, and propensity score matching was done to balance covariates between cohorts. Two-sided p-values <0.05 were considered statistically significant, and analyses adhered to established statistical reporting guidelines.

## Results

After propensity score matching, 308,246 women aged 15 to 45 years who underwent delivery were included, with 154,123 in both the nicotine dependence group and the control group. The matched cohorts were well-balanced across demographic characteristics, including current age, age of delivery, sex, race, and mental, behavioral, and neurodevelopmental disorders (Table 1). Despite overall good covariate balance after propensity score matching, bipolar disorder remained more prevalent in the nicotine dependence cohort. A total of 4,047/154,123 patients (2.6%) in the nicotine dependence cohort and 1,787/154,123 patients (1.2%) in the control group were excluded because they had postpartum psychosis prior to the observation window, leaving 150,076 and 152,336 patients in the nicotine dependence and control groups, respectively (Table 2). During the 60-day postpartum follow-up period, postpartum psychosis occurred in 158/150,076 patients (0.11%) in the nicotine dependence cohort and 70/152,336 patients (0.046%) in the control cohort, corresponding to an absolute risk increase of approximately 0.06 percentage points. Despite the low absolute event rate, nicotine dependence was associated with a significantly increased relative risk of postpartum psychosis (RR, 2.29; 95% CI, 1.73-3.04; p < 0.001) (Table 3).

**Table 1:** Baseline demographics after propensity score matching of nicotine dependence (Cohort 1) and control group (Cohort 2) Continuous variables are presented as mean ± standard deviation (SD) and categorical variables as number (n) and percentage (%). P-values were calculated using the independent-samples t-test for continuous variables and the Pearson chi-square test for categorical variables. Standardized differences (Std Diff) are reported to assess covariate balance after propensity score matching. Statistical significance was defined as p < 0.05.

Characteristic	Nicotine Dependence (Cohort 1)	Control Group (Cohort 2)	P-Value	Std Diff
Current Age (mean ± SD)	33.5 ± 6.0	33.3 ± 6.1	<0.001	0.042
Age at Index (mean ± SD)	27.4 ± 5.5	27.3 ± 5.6	<0.001	0.024
Female, n (%)	154,123 (100%)	154,123 (100%)	—	—
Black or African American, n (%)	37,567 (24.4%)	40,515 (26.3%)	<0.001	0.044
White, n (%)	95,737 (62.1%)	92,606 (60.1%)	<0.001	0.042
American Indian or Alaska Native, n (%)	1,681 (1.1%)	1,738 (1.1%)	0.327	0.004
Asian, n (%)	1,841 (1.2%)	1,800 (1.2%)	0.494	0.002
Native Hawaiian or Other Pacific Islander, n (%)	1,623 (1.1%)	1,698 (1.1%)	0.191	0.005
Other Race, n (%)	5,931 (3.8%)	5,999 (3.9%)	0.525	0.002
Unknown Race, n (%)	9,743 (6.3%)	9,767 (6.3%)	0.859	0.001
Not Hispanic or Latino, n (%)	112,084 (72.7%)	111,981 (72.7%)	0.677	0.001
Hispanic or Latino, n (%)	14,417 (9.4%)	14,492 (9.4%)	0.643	0.002

**Table 2:** Baseline psychiatric diagnoses after propensity score matching of nicotine dependence (Cohort 1) and control group (Cohort 2) Data are presented as number (n) and percentage (%). P-values were calculated using the Pearson chi-square test. Standardized differences (Std Diff) are reported to assess covariate balance after propensity score matching. Statistical significance was defined as p < 0.05.

Diagnosis	Nicotine Dependence Cohort 1, n (%)	Control Group Cohort 2, n (%)	P-Value	Std Diff
Mental, Behavioral, and Neurodevelopmental Disorders	120,416 (78.1%)	120,396 (78.1%)	0.931	<0.001
Depressive Episode	47,657 (30.9%)	53,709 (34.8%)	<0.001	0.084
Bipolar Disorder	13,757 (8.9%)	6,190 (4.0%)	<0.001	0.201
Schizophrenia	1,485 (1.0%)	533 (0.3%)	<0.001	0.077

**Table 3:** Postpartum psychosis risk analysis and statistical measures after propensity score matching A total of 4,047 patients in Cohort 1 and 1,787 patients in Cohort 2 were excluded from the results because they had the outcome prior to the time window. Data are presented as number (n), percentage (%), risk, risk difference, risk ratio, and 95% confidence intervals (CI). P-values were calculated using the chi-square test for comparison of proportions. Statistical significance was defined as p < 0.05.

Cohort	Patients with outcome (postpartum psychosis), n%	Risk	Risk Difference (95% CI)	Risk Ratio (95% CI)	P
Nicotine Dependence (1) n=150,076	158 (0.11%)	0.0011	0.001 (0.00046, 0.00105)	2.291 (1.729, 3.035)	<0.001
Control (2) n=152,336	70 (0.046%)	0.00046			

The proportional hazards assumption was satisfied for the primary outcome, supporting the use of the Cox proportional hazards model (χ² = 1.11, df = 1, p = 0.29). Survival analysis demonstrated a significantly higher hazard of postpartum psychosis among patients with nicotine dependence compared with controls (HR, 2.27; 95% CI, 1.71-3.01; log-rank χ² = 34.42, df = 1, p < 0.001) (Table 4). Kaplan-Meier analysis demonstrated an earlier and greater cumulative incidence of postpartum psychosis among patients with nicotine dependence throughout the 60-day postpartum follow-up period (Tables 4, 5). At the end of follow-up, the survival probability was 99.89% (149,918/150,076 patients) in the nicotine dependence cohort and 99.95% (152,266/152,336 patients) in the control cohort, indicating a very low absolute event rate despite the significantly higher relative hazard associated with nicotine dependence.

**Table 4:** Statistical measures after propensity score matching Data are presented as hazard ratios (HRs) with 95% confidence intervals (CIs) and log-rank test statistics (χ², degrees of freedom (df), and p-values). The log-rank test was used to compare Kaplan-Meier survival curves between cohorts, and the Cox proportional hazards model was used to estimate the hazard ratio for postpartum psychosis. The proportional hazards assumption was assessed using the Schoenfeld residuals test (χ² = 1.11, df = 1, p = 0.29). Statistical significance was defined as p < 0.05.

Test / Estimate	Value	95% CI	χ²	df	p
Log-Rank Test	34.422	—	34.422	1	<0.001
Hazard Ratio (Proportionality)	2.269	(1.712, 3.006)	1.11	1	--

**Table 5:** Postpartum psychosis Kaplan-Meier analysis after propensity score matching Data are presented as total cohort size (n), patients with outcome (n), and Kaplan-Meier survival probability (%). Survival curves were compared using the log-rank test, and hazard ratios were estimated using a Cox proportional hazards model. Statistical significance was defined as p < 0.05.

Cohort	Patients in Cohort	Patients With Outcome	Survival Probability (%)
Nicotine Dependence (1)	150,076	158	99.89
Control (2)	152,336	70	99.95

## Discussion

In this large retrospective cohort study of more than 300,000 women who underwent delivery, nicotine dependence was associated with a significantly increased risk of postpartum psychosis within 60 days of childbirth. After propensity score matching for demographic characteristics and psychiatric comorbidity, individuals with nicotine dependence experienced more than double the risk of developing postpartum psychosis compared with controls (risk ratio, 2.29; hazard ratio, 2.27). While the absolute event rate remained low in both groups, nicotine dependence was associated with a higher risk across all risk estimates, lending clinical credibility to the observed association. This finding was further supported by time-to-event analysis demonstrating earlier and greater cumulative incidence among nicotine-dependent patients. Together, these findings suggest that nicotine dependence is a clinically relevant risk marker associated with both the likelihood and timing of postpartum psychosis, even in the setting of a low absolute event rate.

Mechanistic studies suggest that interactions between estrogen signaling, nicotinic cholinergic pathways, and hypothalamic-pituitary-adrenal (HPA) axis dysregulation contribute to vulnerability to postpartum mood disorders, potentially linking smoking behavior to depressive symptoms in a subset of women [8]. Given the profound hormonal and stress-axis dysregulation that characterizes postpartum psychosis, similar neuroendocrine mechanisms may plausibly influence susceptibility to psychotic symptoms in the postpartum period. However, further studies are needed to elucidate the mechanisms underlying the association between nicotine dependence and postpartum psychosis.

To our knowledge, this is the first large population-based study to evaluate the association between nicotine dependence and postpartum psychosis. By extending prior literature linking nicotine exposure to postpartum depression and psychotic illness outside the perinatal period [4-6], our findings identify nicotine dependence as a potential risk marker for postpartum psychiatric illness. Our results parallel prior studies, suggesting that nicotine dependence may be associated more broadly with postpartum psychiatric instability rather than with mood symptoms alone.

From a clinical standpoint, even a rare outcome of postpartum psychosis warrants attention when associated with high morbidity. The need for psychiatric hospitalization, driven by the increased risk of maternal suicide and infanticide, underscores the critical clinical importance of monitoring and understanding rates of postpartum psychosis. Postpartum psychosis not only represents an acute psychiatric emergency, but longitudinal studies show a high rate of recurrent mood or psychotic episodes independent of childbirth, especially within the bipolar spectrum, indicating potentially worse long-term outcomes compared with postpartum depression [9]. The more than twofold elevation in relative risk observed here suggests that pregnant patients with nicotine dependence may benefit from anticipatory guidance, earlier follow-up, and heightened vigilance for emergent psychotic symptoms in the immediate postpartum period. Integration of obstetric and psychiatric care, particularly for individuals with co-occurring mental health histories, may be especially valuable.

Strengths and limitations

This study should be interpreted in light of limitations inherent to retrospective database analyses, and residual confounding cannot be excluded. Postpartum psychosis diagnoses were identified using ICD-10-CM codes, which may be subject to misclassification. In particular, the broad F20-F29 category includes psychotic disorders that are not specific to postpartum psychosis. However, restricting qualifying diagnoses to the 1- to 60-day period following delivery and excluding patients with these diagnoses prior to the outcome window increased the specificity of the outcome definition for new-onset postpartum presentations. Detailed information on smoking intensity, duration, or cessation during pregnancy was unavailable, precluding dose-response analyses and limiting insight into critical exposure windows. We were unable to exclude individuals with pre-existing mood disorders prior to pregnancy, as doing so substantially reduced the sample size. Importantly, bipolar disorder remained more prevalent in the nicotine dependence cohort after propensity score matching (SMD = 0.201), and residual differences in schizophrenia spectrum disorders were also present. Given the strong association of these psychiatric conditions with postpartum psychosis, these imbalances may have contributed to the observed association and represent important sources of residual confounding. Additional adjustment or sensitivity analyses addressing these residual imbalances were not performed. Furthermore, unmeasured or incompletely captured factors, including socioeconomic status, other substance use, psychosocial stressors, and healthcare utilization, may differ between cohorts and may partially contribute to the observed association. Given the potential for residual confounding despite propensity score matching, the observed association should not be interpreted as evidence of a causal relationship between nicotine dependence and postpartum psychosis. Additionally, the rarity of postpartum psychosis means that small differences in case ascertainment may influence absolute event rates.

Notwithstanding these limitations, key strengths include the large, diverse population, real-world clinical setting, and propensity score matching strategy, which together allowed evaluation of the association across multiple analytic approaches. The concordance between relative risk and time-to-event estimates, along with confirmation of proportional hazards assumptions, supports the internal validity of the findings. Future research should evaluate whether the timing of nicotine exposure, specifically pre-pregnancy versus prenatal smoking, differentially influences postpartum psychosis risk and should further investigate neuroendocrine mechanisms linking nicotine exposure to acute postpartum psychiatric vulnerability. Stratified analyses by specific psychiatric diagnoses, including major depressive disorder, bipolar disorder, psychotic disorders, and schizophrenia, may further refine risk estimates for postpartum psychosis and represent an important next step for future research. Prospective studies are needed to better understand biological vulnerability and improve early identification of postpartum psychosis.

## Conclusions

Nicotine dependence was associated with a significantly increased risk of postpartum psychosis in the early postpartum period. Recognition of this relationship may help clinicians identify patients at elevated risk and facilitate earlier monitoring and intervention during the high-risk postpartum period.
